# Comparative Investigation of the Nutritional Profiling and Antipyretic Activity of *Moringa oleifera* Leaves, Bark, and Root From Different Sites of Punjab, Pakistan

**DOI:** 10.1002/fsn3.4706

**Published:** 2024-12-23

**Authors:** Shaista Jamil, Tanveer Hussain Turabi, Saeed Ahmad, Muhammad Riaz, Hafiz Muhammad Wariss, Quzi Sharmin Akter

**Affiliations:** ^1^ Institute of Forest Sciences, Faculty of Agriculture and Environment The Islamia University of Bahawalpur Bahawalpur Pakistan; ^2^ Office of Research, Innovation and Commercialization (ORIC) University of Sargodha Sargodha Pakistan; ^3^ Department of Allied Health Sciences University of Sargodha Sargodha Pakistan; ^4^ Department of Botany University of Sargodha Sargodha Pakistan; ^5^ CAS Key Laboratory of Biogeography and Bioresource in Arid Land Xinjiang Institute of Ecology and Geography Urumqi People's Republic of China; ^6^ Department of Genetics and Animal Breeding, Faculty of Animal Science and Veterinary Medicine Patuakhali Science and Technology University Patuakhali Bangladesh

**Keywords:** antipyretic activity, *Moringa oleifera*, proximate analysis

## Abstract

The utilization of various *Moringa oleifera* plant sections as a medicinal and nutritional source for humans and animals has been the subject of significant research in recent years. This study aimed to investigate the nutritional profiling through proximate analysis and the antipyretic activity of *M. oleifera* leaves, bark, and root in methanolic extract from different sites of Punjab, Pakistan. Methanolic extract of leaves, bark, and root from sites i to e of Southern Punjab, Central Punjab, and Northwest Punjab as S1, S2, and S3, respectively, at doses of 50, 100, and 150 mg/kg bw showed statistically significant results as compared to the positive and negative controls. The S1 leaves showed marvelous proximate compositions and antipyretic activity as a result of significantly lowering temperature as compared to the other methanolic leaves, bark, and root extracts of *M. oleifera* plant sample collected from other sites. The antipyretic activity of *M. oleifera* leaves, bark, and root was investigated using the standard reference drug paracetamol (200 mg/kg). The antipyretic activity was evaluated using baker's yeast‐induced pyrexia. Obtained data were analyzed using one‐way ANOVA followed by Tukey's post hoc test and *p <* 0.05 was considered significant. The study showed that the methanolic extract of *M. oleifera* leaves possesses highest antipyretic activities as compared to bark and root which justifies its use as nutritional and traditional medicine in the treatment of fever.

## Graphical abstract

Leaves of *Moringa oleifera* have highest nutritional content compared to the bark and roots. It was found that S1 leaves, bark, and root extract of *M. oleifera* Lam demonstrated maximum antipyretic activity when compared to the S2 and S3 leaves, bark, and root extracts. The S1 leaves extract demonstrated outstanding antipyretic outcomes for reducing rectal temperature in rabbits. Antipyretic activity of S1 methanolic leaves extract of *M. oleifera* on *Escherichia coli* induced pyrexia in rabbits. S1 = Bahawalpur, NC = negative control, PC = positive control (paracetamol).

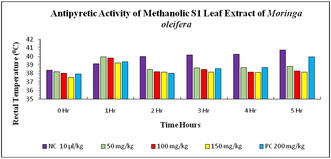

## 1 Introduction

*Moringa oleifera* belong to the family Moringaceae (Kunwar, Jain, and Verma 2022, Singh et al. 2022) which necessitate 13 species including *M. oleifera* , *Moringa arborea*, *Moringa borziana*, *Moringa concanensis*, *Moringa hildebrandtii*, *Moringa peregrina*, *Moringa rivae*, *Moringa pygmaea*, *Moringa ovalifolia*, *Moringa stenopetala*, *Moringa ruspoliana*, *Moringa drouhardii*, and *Moringa longituba* (Farooq and Koul 2020; Kunwar, Jain, and Verma 2022; Jia et al. 2023). The Miracle Tree identified as *M. oleifera* is a member of the Moringaceae family. The plant is locally known as Mulangay, drumstick tree, horseradish tree, benzolive, mlonge, marango, saijihan, sajna, and kelor (Kumar et al. 2022; Jia et al. 2023; Pareek et al. 2023).

*Moringa oleifera* plant grow at temperatures between 25°C and 40°C and between 250 and 300 mm of yearly precipitation. It may live on soil that is ascetically alkaline and sandy or loamy. A fast emerging deciduous tree *M. oleifera* grows to a height of 10–12 m and up to 45 cm girth (Shantha, 2022; Arif, Bajguz, and Hayat 2023). Plants exhibited a speckled range of pharmacological and therapeutic properties including antitumor, anticancer, antipyretic, anti‐inflammatory, antiulcer, antifungal, and antidiabetic. In addition to these pharmacological and therapeutic properties, local peoples use *M. oleifera* Lam to treat cardiac issues (Shivangini, Mona, and Nisha 2022). All parts of the *M. oleifera* Lam sections have demonstrated virtuous pharmacological activities in achieving the therapeutic benefits that boost up the immune system (Padma et al. 2022; Padma, Jagadish, and Singh 2022). It is also surprising how well the tree can lessen the effects of climate change. Because of its ability to adapt weather, soil, and other environmental conditions, *M. oleifera* is also known as the “never‐die” plant even in the dry season. The tree's heavy flushes serve as a good sink for absorbing and using carbon dioxide, hence lowering the quantity of atmospheric carbon dioxide. One of the main causes of ozone layer depletion and global warming is the disruption in carbon dioxide. In order to survive these threats from food insecurity, *M. oleifera* tree is a crop that can adapt to climate change (Walia, Kaur, and Sharma 2022).

In Pakistan's southern Punjab, the perennial plant *M. oleifera* Lam is arguably the most widely planted type there. The history reveals that medicinal herbs have been used for centuries. Evidence of herbal medicine dating back to Nagpur around 5000 years ago has been discovered. Worldwide medicinal plants are used to treat an assortment of ailments (Patil et al. 2022). *Moringa oleifera* extracts serve as a low‐cost biostimulant, environment friendly, and bioenhancer that promotes sustainable crop development and agricultural operations. The chemical substances derived from plants are known as phytochemicals. The chemical components of plants have defensive and disease‐preventive properties but are not nutritional. These plant chemicals support the smell, color, flavor, and texture of plants as well as the influence they have on human health (Adusei et al. 2022). Numerous pharmacological solicitations can be made from the range of phytochemicals found in the *M. oleifera* species (Abd Rani, Husain, and Kumolosasi 2018). This study was planned to explore and compare the nutritional profiling and antipyretic activity of different parts of *M. oleifera* collected from Bahawalpur, Faisalabad, and Mianwali, Punjab, Pakistan.

## 2 Materials and Methods

### 2.1 Collection of Plant Material and Extract Preparation

The current research work was planned to study the nutritional profiling and antipyretic activity of methanolic extract of *M. oleifera* Lam leaves, bark, and root collected from Southern Punjab region of Bahawalpur adjoining area of Cholistan desert (site area 1), Central Punjab city Faisalabad (site area 2), and the Northwest region of Punjab city Mianwali (site area 3), abbreviated as S1, S2, and S3, respectively. The selected plant parts were collected during the month of March from South and Central Punjab, while in April from Northwest Punjab. The collected plants parts were identified and authenticated by a taxonomist from the Department of Botany of the same university and the voucher specimens were submitted under the voucher numbers 329/Botany/IUB, 330/Botany/IUB, and 321/Botany/IUB for S1, S2, and S3, respectively.

The collected plant materials were shade dried and then grinded into fine powder with an electric mill and stored in airtight container. Powdered plant sample were placed in bucket and soaked in methanol for 7 days. The soaked plant materials were filtered with the help of Whatman no. 1 filter paper. The obtained methanolic filtrates were evaporated using rotary evaporator and concentrated extracts were obtained. The crude plant extracts were collected in airtight glass container to avoid contamination.

### 2.2 Determination of Moisture

Moisture content was determined by following the method of de Mello et al. (2020). For moisture analysis, an empty dry Petri dish (W1) was weighed, and after taring, added 10 g of plant sample (W2). The Petri dish was then placed in a hot air oven set at 105°C for 24 h. The Petri dish containing the sample was placed in a desiccator for 5 min and was weighed again (W3). The weight difference was evaluated by the following formula:

$$\mathrm{DM}\%=\left(\text{W3}-W1/\text{W2}\right)\times 100$$

### 2.3 Determination of Ash

AOAC technique was used to determine the ash content of the plant samples (Thiex et al. 2012, de Mello et al. 2020). A total of 2 g sample was taken, weighed, and then added to a dry empty crucible (W1) that has been baked in an oven to eliminate the moisture; subsequently recorded the weight (W2). The sample is heated on a hot plate until the vapors are gone, then it is placed in a muffle furnace and heated to 650°C for 3 h. The heated crucible weighed with the sample and desiccated to bring it to room temperature (W3).

$$\mathrm{Ash}\%=\left(\text{W3}-W1/\text{W2}\right)\times 100$$

### 2.4 Determination of Crude Protein (CP)

A Kjeldhal digestion flask was filled with 1 g oven‐dried plant sample. In the digestion flask, 5 g of the digestion mixture and 25 mL of concentrated sulfuric acid were added. The digestion flask was then placed at 420°C for 4–6 h, the rack was placed in the digestion block heater underneath the fume hood, and the exhaust manifold linked to the water aspirator was installed. The exhaust manifold rack was taken out of the digester and placed under the fume hood to cool at room temperature. Move the flasks to the distillation apparatus. Place the digested sample in a 250‐mL flask and then add distilled water to make the volume up to 250 mL. To get rid of the entire digested material, wash the flask three times with distilled water. Put a 10‐mL sample in the distillation apparatus, take 10 mL of 40% NaOH, and 10 mL of water should be added to the distillation apparatus. Then, 10 mL of boric acid was added in a different flask followed by the addition of one drop of indicator and position it underneath the distillation equipment. Condensed liquid should be collected in a conical flask with indicator solution until the color turns light straw and the volume reaches 25 mL. Titrate with sulfuric acid against N/1000 until the color turns light pink. Note the acid volume utilized (Horwitz, 1975).

$$\begin{array}{c} \text{Crude protein}\%=(D\times 0.00014\times V/W\times \\ \;\text{Volume of}\;40\%\text{NaOH used})\times 100 \end{array}$$

### 2.5 Crude Fiber

The crude fiber content was assessed by using Fibertec equipment. Taken 2 g of fat‐free sample (W) into clean crucible and set up on a crucible stand. To get rid of any possible fats, the samples were cleaned using acetone. Following the crucibles placement in the apparatus, hot extraction was carried out repeatedly in 150 mL of each of the 1.25% potassium hydroxide and sulfuric acid solutions for 30 min. Following extraction, the samples were cleaned three times in acetone before being dried for 2 h at 130°C. The samples were dehydrated, ventilated at room temperature, and weighed. Then, crucible was placed in muffle furnace at 525°C for 3 h (Commission, E. 2009; Pushpakumara et al. 2023).

$$\text{Crude fiber}\%=\left(\text{W2}-\text{W3}-C/\text{W1}\right)\times 100$$

### 2.6 Organic Matter

The organic matter % was calculated by subtracting the % moisture and % ash from 100 (Sultana 2020).

$$\text{Organic matter}\%=100-\left(\%\text{moisture}+\%\mathrm{ash}\right)$$

### 2.7 In Vivo Study for Antipyretic Activity

In order to conduct the studies, adult healthy 2 months old male rabbits, weighing 1.5–2.0 kg with a rectal temperature of 39°C ± 0.5°C of the native strain *Oryctolagus cuniculus* were acquired from a local market in Bahawalpur and then housed individually in separate cages. The baseline body temperature was measured at a fixed time every morning. The laboratory temperature was maintained between 22°C and 25°C, with a humidity of 40%–60%.

### 2.8 Preparation of *Escherichia coli* Suspension

*Escherichia coli* pure and identifiable cultures were obtained on MacConkey agar from the Microbiology Laboratory, Quaid‐e‐Azam Medical College, Bahawalpur and incubated at 37°C for 24 h. Colony counter was used to tally the colonies. One colony was selected, cleaned in standard saline, placed on agar plates for reculturing, and incubated for 24 h. The number of organisms in one drop was multiplied by the quantity of drops in 1 mL to determine the overall number of organisms. There were 127 × 10^7^ *E. coli* in 1 mL of fluid.

### 2.9 Induction of Pyrexia in Experimental Animals

*Escherichia coli* suspension was injected into a rabbit's marginal ear vein at a concentration of 0.01 mL per kg body weight to induce fever in the animals (Ahmad et al. 2014). Prior to and during the injection of *E. coli* , the rectal temperature was measured using a digital thermometer at regular intervals throughout the experiment.

### 2.10 Drug Administration

*Escherichia coli* suspension was injected into the animal for 1–2 h before pyrexia developed. Plant extracts in methanol were employed at 50, 100, and 150 mg/kg bw doses.

### 2.11 Study Protocol

Digital thermometers were used to take the rabbits' rectal temperatures at 0 h before injecting *E. coli* solution. During the 1‐h interval, take a digital rectal temperature reading once more. The treatment group was administered with the plant extracts of leaves, bark, and root, while the positive control group received paracetamol orally. For 4 h, the rectal temperature was taken once per hour. Each of the five groups comprised five rabbits each and were utilized for the evaluation of each plant portion. The administration of paracetamol (200 mg/kg bw) to two groups served as both a positive control and a negative control.

### 2.12 Antipyretic Activity of *M. oleifera* Lam

Fever in rabbits was induced by injecting the *E. coli* suspension in the marginal ear of the rabbits at the concentration of 0.01 mL per kg body weight (Riffat, S. et al., 1982). The animals having temperature were divided into five groups, having five animals in each group and treated orally as follows:

Group 1: Positive control (given *E. coli* suspension).

Group 2: Negative control (given paracetamol 200 mg/kg).

Group 3: Treatment group 1 (methanolic plant extract 50 mg/kg bw).

Group 4: Treatment group 2 (methanolic plant extract 100 mg/kg bw).

Group 5: Treatment group 3 (methanolic plant extract 150 mg/kg bw).

By using protocols recommended by the Association of Analytical Chemists, the leaves protein concentrates approximate composition was calculated (Horwitz 1975). Numerous categories of nutrients included in the plant sample such as moisture, dry matter, ash, fat, crude fiber, carbohydrates, and calorific value were determined to make up the sample's proximate composition. The proximate analysis dappled in the laboratory of Cholistan Institute of Desert Studies, The Islamia University, Bahawalpur and the Laboratory of Food Science and Nutrition, Bahauddin Zakariya University, Multan.

## 3 Results and Discussion

### 3.1 Proximate Analysis of *M. oleifera* Leaves, Bark, and Root

The leaves, bark, and root were analyzed for moisture content, ash, crude fat, crude fiber, crude protein, and organic matter. The results are given in Table 1. The highest moisture content was recorded in S3 leaves (20.02%), followed by S2 (19.61%), and lowest value in S1, which was 16.34%, as lesser moisture corresponds with higher dry mass. The highest ash content was observed in S1 (7.72%), followed by S2 (7.41%), whereas S3 exhibited lowest ash content (7.35%). The leaves of S1 showed significant amount of crude fat (3.58%), followed by S2 (3.38%) and S3 (3.23%). The crude fiber content was found highest in S1 (8.47%), followed by S2 (7.35%), whereas lowest crude fiber was recorded in S3 (7.15%) leaves powder of *M. oleifera* . The crude protein was also recorded highest (9.84%) in S1, followed by 8.88% in S2, and least (8.75%) in S3. The peak value (75.94%) of organic matter was in S1 and 72.97% in S2, while 72.62% in S3 leaves powder of *M. oleifera* (Table 1). The maximum percentage (17.44%) of moister content was documented in S3, followed by S2 (16.98%), and lowest value (16.76%) of moisture content in S3 bark powder of *M. oleifera*. The highest percentage of ash, crude fat, crude fiber, and crude protein were 41.17%, 4.28%, 41.21%, and 7.61%, respectively, in S1 bark powder of *M. oleifera*. This plant exhibited the percentage of ash, crude fat, crude fiber, and crude protein as 32.07%, 4.10%, 36.64%, and 5.46%, respectively, in S2 bark of *M. oleifera*, while 33.03%, 4.04%, 34.18%, and 6.56% in S3 bark powder of *M. oleifera* . The maximum content of organic matter was recorded in S2 (50.59%) and S3 (50.21%), and least value was recorded in S1 (41.35%) powdered bark of *M. oleifera* (Table 1). The root of *M. oleifera* showed 15.86% moisture content, 58.17% ash, 5.13% crude fat, 57.68% crude fiber, and 5.87% crude proteins, while organic matter was 25.97% in S1. The S2‐powdered root sample of *M. oleifera* exhibited moisture content as 16.29%, ash as 47.66%, crude fat as 4.82%, crude fiber as 58.14%, crude proteins as 4.38%, and organic matter as 36.03%, whereas the S3 showed moisture content as 16.07%, ash as 49.44%, crude fat as 4.72%, crude fiber as 55.76%, crude proteins as 4.38%, and organic matter as 34.50% (Table 1). Potisate, Kerr, and Phoungchandang (2015) evaluated the proximate composition of *M. oleifera* leaves at 15°C–35°C during storage of dried leaves in propylene packaging and reported the storage time as significant factor for moisture content. They observed no significant change in the moisture content in samples stored at lower temperature (15°C), while higher moisture content (10.32%) was observed in samples stored at 35°C in propylene packaging (Potisate, Kerr, and Phoungchandang 2015). Al Juhaimi et al. (2017) compared the mineral contents and oxidative status of different Moringa species including *M. oleifera* , *M. peregrina*, and *Sonchus oleraceus* . Their study results indicated *S. oleraceus* leaves possess high mineral content and antioxidant compounds as compared to Moringa species. A published study reported an appreciable amount of oil, moisture content, ash content, crude fiber, and carbohydrates serving as an important source of nutrients in *M. oleifera* leaves and seeds. Additionally, the leaves and seeds of *M. oleifera* can contribute remarkably to the nutritional requirements and the management of human health (Özcan 2020; Al Juhaimi et al. 2016).

**TABLE 1:** Proximate composition of *Moringa oleifera* leaves, bark, and roots from selected study sites.

*M. oleifera* part used	Site area	Moisture contents %	Ash %	Crude fat %	Crude fiber %	Crude protein %	Organic matter %
Leaves	S1	16.34 ± 0.04^c^	7.72 ± 0.03^a^	3.58 ± 0.04^a^	8.47 ± 0.05^a^	9.84 ± 0.04^a^	75.94 ± 0.06^a^
	S2	19.61 ± 0.03^b^	7.41 ± 0.06^b^	3.38 ± 0.05^b^	7.35 ± 0.05^b^	8.88 ± 0.07^b^	72.97 ± 0.02^b^
	S 3	20.02 ± 0.05^a^	7.35 ± 0.07^b^	3.23 ± 0.06^c^	7.15 ± 0.03^c^	8.75 ± 0.04^c^	72.62 ± 0.12^c^
Bark	S1	16.76 ± 0.03^b^	41.17 ± 0.06^a^	4.28 ± 0.03^a^	41.21 ± 0.54^b^	7.61 ± 0.07^a^	41.35 ± 0.02^c^
	S2	16.98 ± 0.02^a^	32.07 ± 0.06^c^	4.10 ± 0.04^b^	36.64 ± 0.05^a^	5.46 ± 0.05^c^	50.59 ± 0.08^a^
	S3	17.44 ± 0.05^a^	33.03 ± 0.05^b^	4.04 ± 0.04^b^	34.18 ± 0.03^b^	6.56 ± 0.05^b^	50.21 ± 0.08^b^
Root	S1	15.86 ± 0.03^c^	58.17 ± 0.05^a^	5.13 ± 0.03^a^	57.68 ± 0.05^a^	5.87 ± 0.05^a^	25.97 ± 0.06^c^
	S2	16.29 ± 0.03^a^	47.67 ± 0.06^c^	4.82 ± 0.04^b^	58.14 ± 0.04^b^	4.38 ± 0.04^b^	36.03 ± 0.04^a^
	S3	16.07 ± 0.04^b^	49.44 ± 0.06^b^	4.72 ± 0.04^b^	55.76 ± 0.04^b^	4.38 ± 0.04^b^	34.50 ± 0.10^b^

*Note:* Values are expressed as the mean ± SED (minimum of triplicate analysis), means in a column not sharing the same letter are significantly different (*p* < 0.05). Site areas: S1 = Bahawalpur, S2 = Faisalabad, S3 = Mianwali.

### 3.2 Antipyretic Activity of *M. oleifera*

**FIGURE 1:**
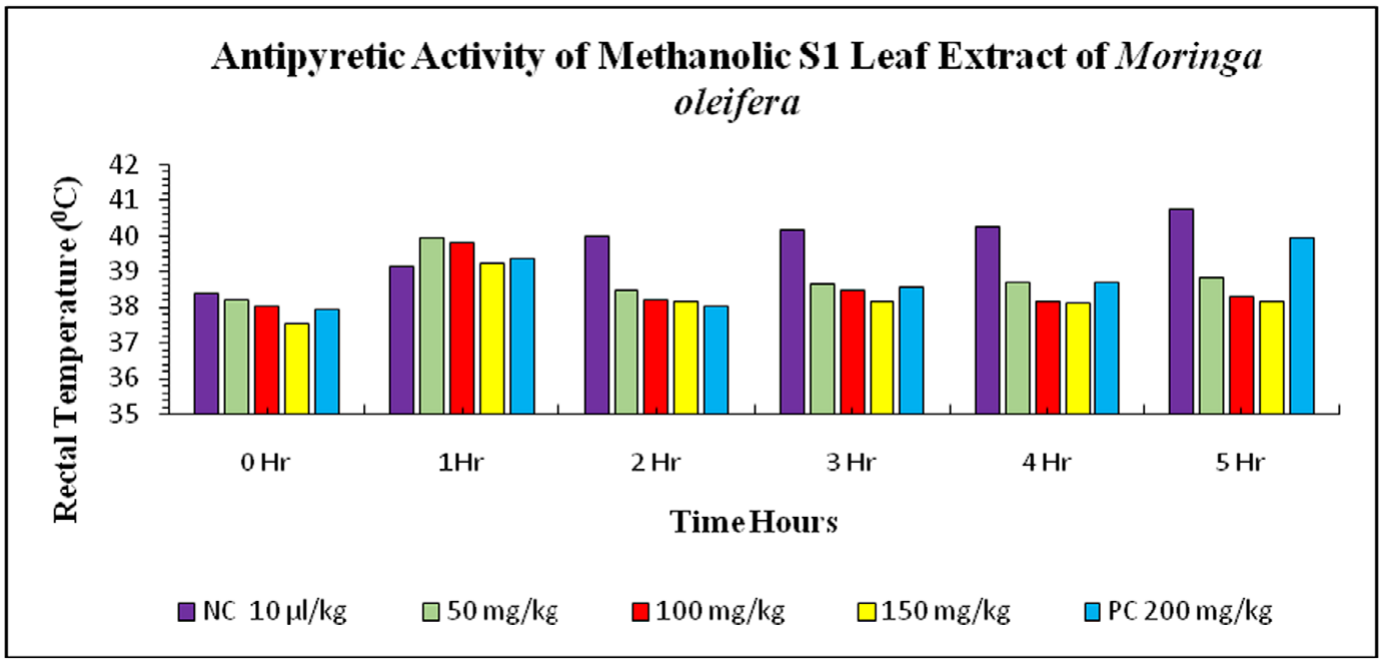
Antipyretic activity of S1 methanolic leaves extract of *Moringa oleifera* on *Escherichia coli‐*induced pyrexia in rabbits. NC = negative control, PC = positive control (paracetamol), S1 = Bahawalpur.

**FIGURE 2:**
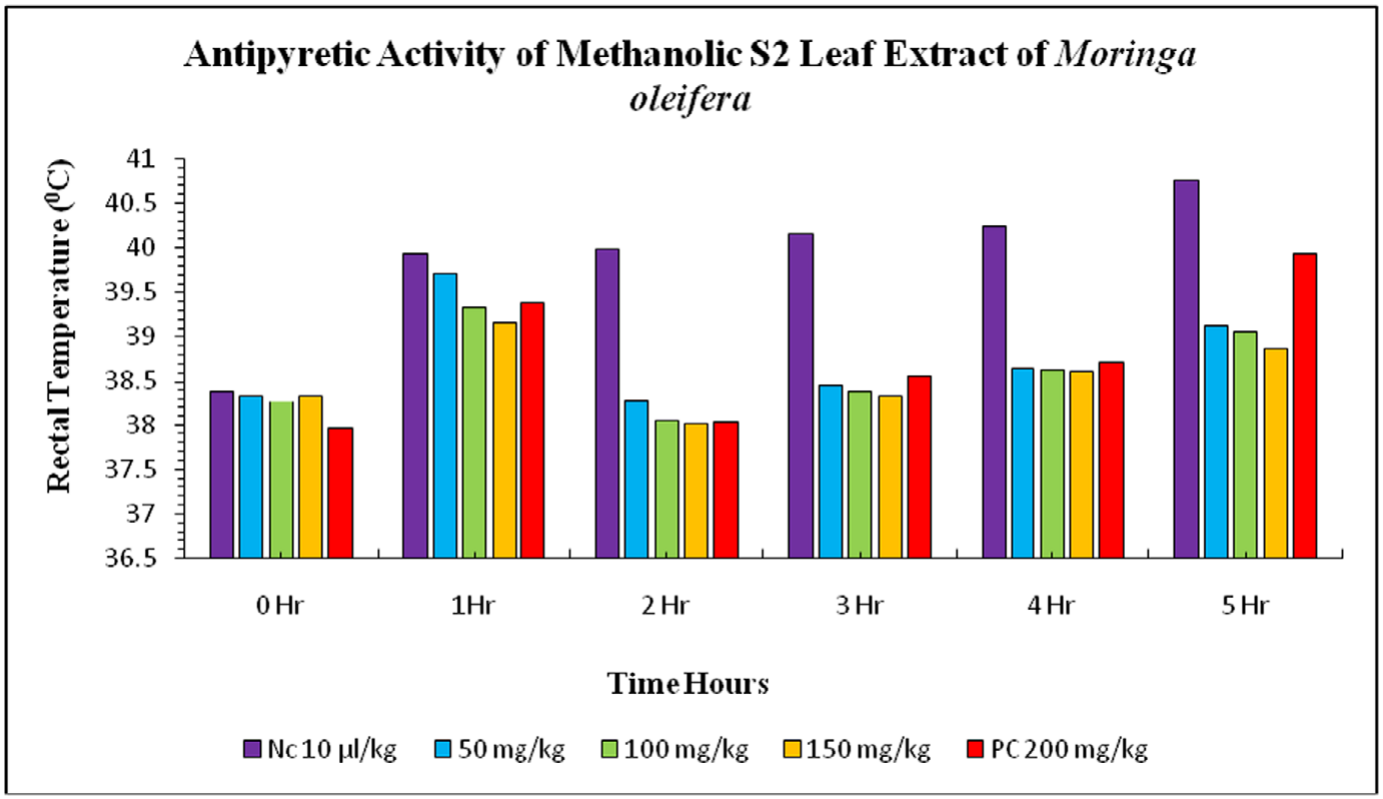
Antipyretic activity of S2 methanolic leaves extract of *Moringa oleifera* on *Escherichia coli* ‐induced pyrexia in rabbits. NC = negative control, PC = positive control (paracetamol), S2 = Faisalabad.

**FIGURE 3:**
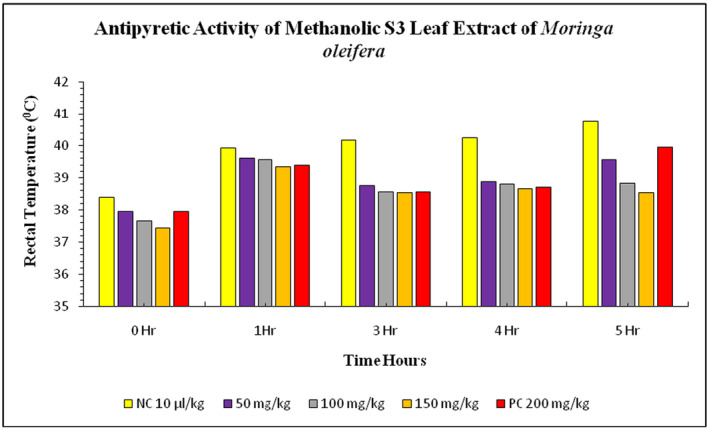
Antipyretic activity of S3 methanolic leaves extract of *Moringa oleifera* on *Escherichia coli‐*induced pyrexia in rabbits. NC = negative control, PC = positive control (paracetamol), S3 = Mianwali.

The results of the effect of methanolic S1 leaves extract of *M. oleifera* in *E. coli* ‐induced febrile rabbits are shown in Figure 1. In rabbits, the initial rectal temperatures before injection of *E. coli* suspension in negative control and positive control groups were 38.39°C ± 0.03°C and 37.96°C ± 0.02°C, respectively. At doses 50, 100, and 150 mg/kg bw treatment group animals, the rectal temperatures were recorded as 38.19°C ± 0.02°C, 38.02°C ± 0.05°C, and 37.54°C ± 0.02°C, respectively. The study involved injecting *E. coli* suspension into rabbits in five groups. The results showed that the rabbits' rectal temperature increased in the negative control group and positive control group, while the treatment group showed a decrease in rectal temperature. The methanolic S1 leaves extract treatment showed a significant dose‐dependent decrease in the rectal temperature in the order of 38.47°C ± 0.03°C at 50 mg/kg, 38.21°C ± 0.03°C at 100 mg/kg, and 38.16°C ± 0.03°C at 150 mg/kg. The maximum decrease in rectal temperature was observed in all doses, indicating dose‐dependent results of the *M. oleifera* S1 leaves extract. The effect of *M. oleifera* methanolic S2 leaves extract on *E. coli* induced pyrexia in rabbits. The normal body temperature before the injection of *E. coli* suspension was 38.39°C ± 0.03°C in the negative control group (distilled water) and 37.96°C ± 0.02°C in the positive control group (paracetamol). After 1 h, the rectal temperature increased gradually to 39.93°C ± 0.02°C in the negative control group and 39.38°C ± 0.02°C in the positive control group. The treatment groups showed a decrease in rectal temperature, reaching up to 38.45°C ± 0.06°C in the 50 mg/kg bw treatment group, 38.39°C ± 0.04°C in 100 mg/kg bw treatment group, and 38.34°C ± 0.05°C in 150 mg/kg bw treatment group. The maximum rectal temperature decreased in the highest dose at 150 mg/kg of S2 leaves extract compared to the negative (distilled water) and positive control groups (paracetamol) (Figure 2). The normal body temperature of rabbits before the injection of *E. coli* suspension was 38.39°C ± 0.03°C. The treatment groups of S3 leaves extract at doses 50, 100, and 150 mg/kg showed a gradual increase in rectal temperature. After 1 h, the rectal temperature increased to 39.93°C ± 0.02°C in the negative control group and 39.38°C ± 0.02°C in the positive control group. The treatment group with methanolic S3 leaves extract also showed a decrease in rectal temperature. The rectal temperature decreased with the dose increases at 50, 100, and 150 mg/kg bw treatments (Figure 3).

**FIGURE 4:**
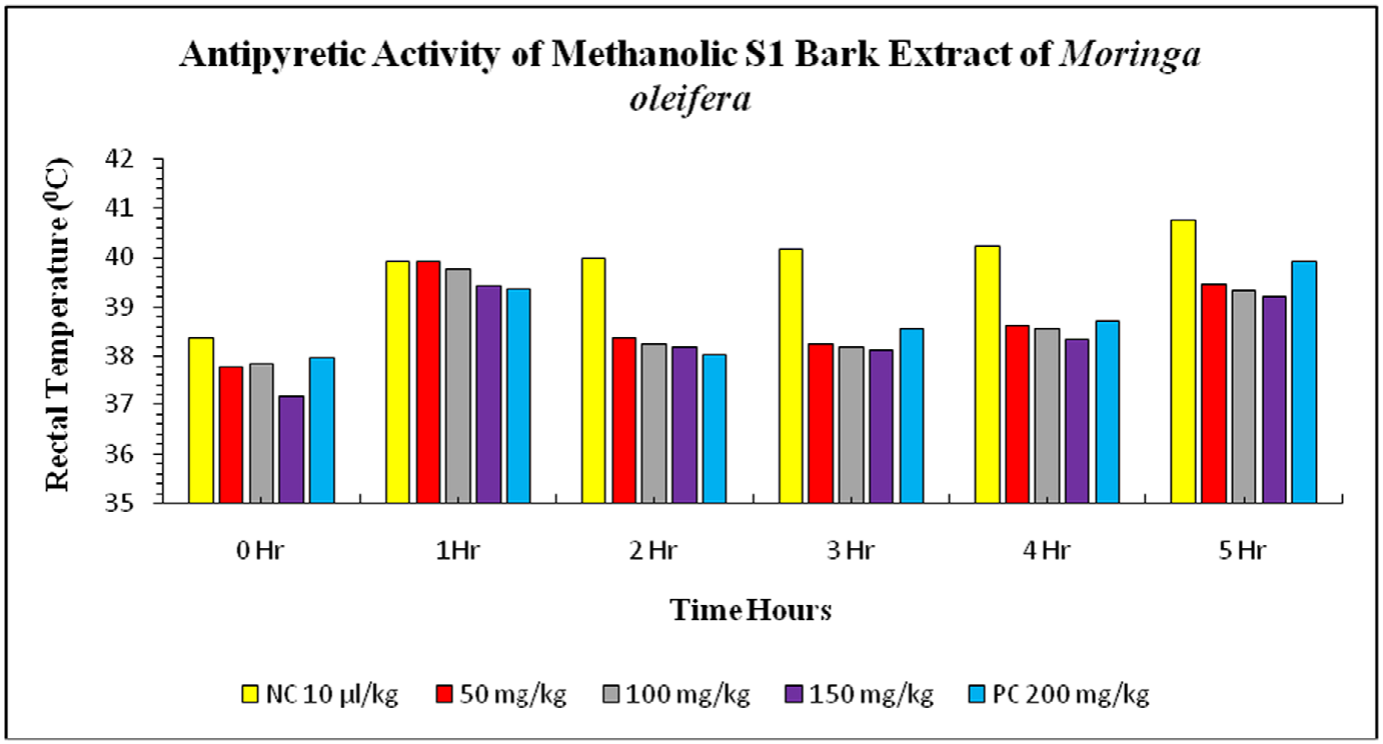
Antipyretic activity of S1 methanolic bark extract of *Moringa oleifera* on *Escherichia coli‐*induced pyrexia in rabbits. NC = negative control, PC = positive control (paracetamol), S1 = Bahawalpur.

**FIGURE 5:**
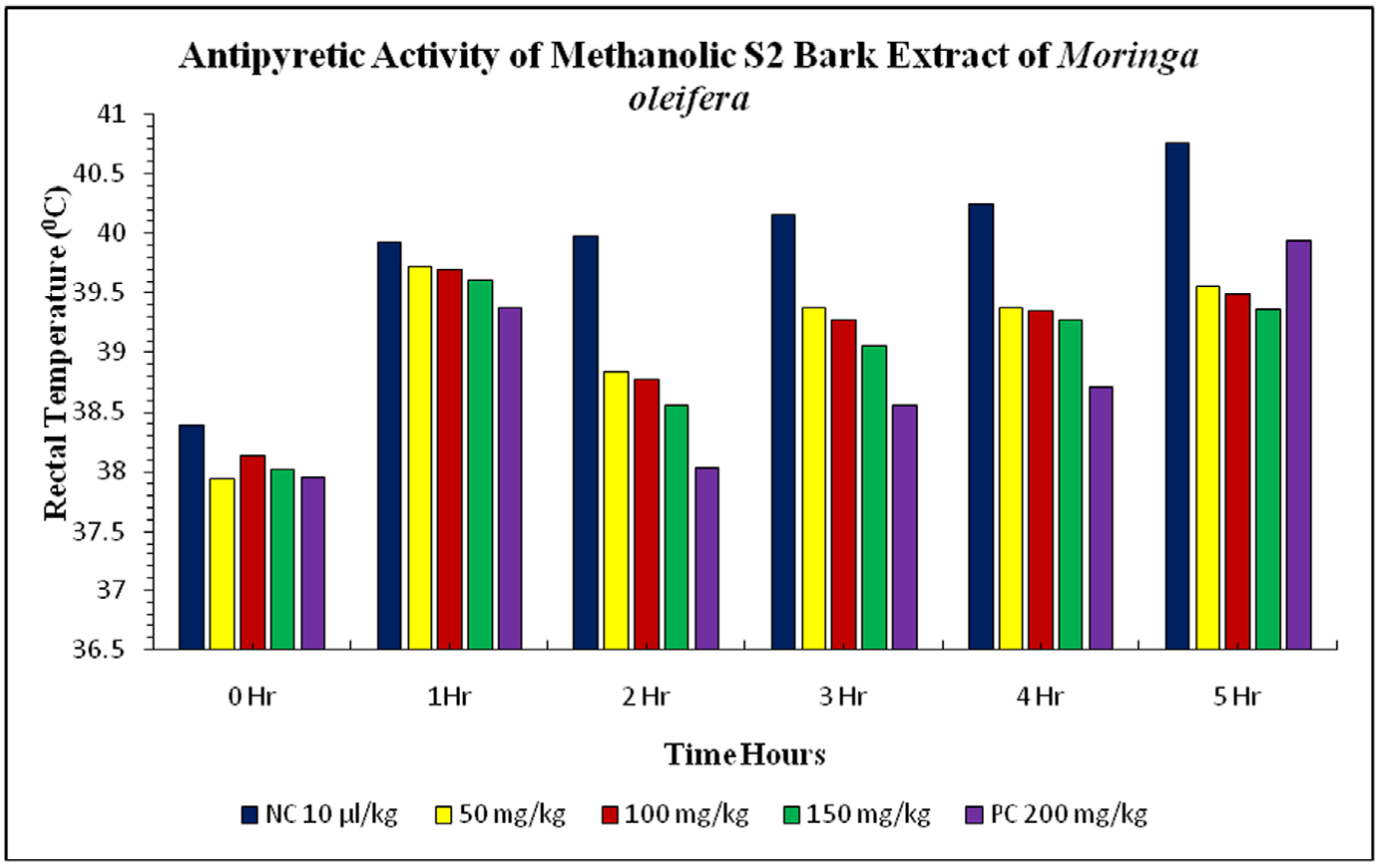
Antipyretic activity of S2 methanolic bark extract of *Moringa oleifera* on *Escherichia coli* ‐induced pyrexia in rabbits. NC = negative control, PC = positive control (paracetamol), S2 = Faisalabad.

**FIGURE 6:**
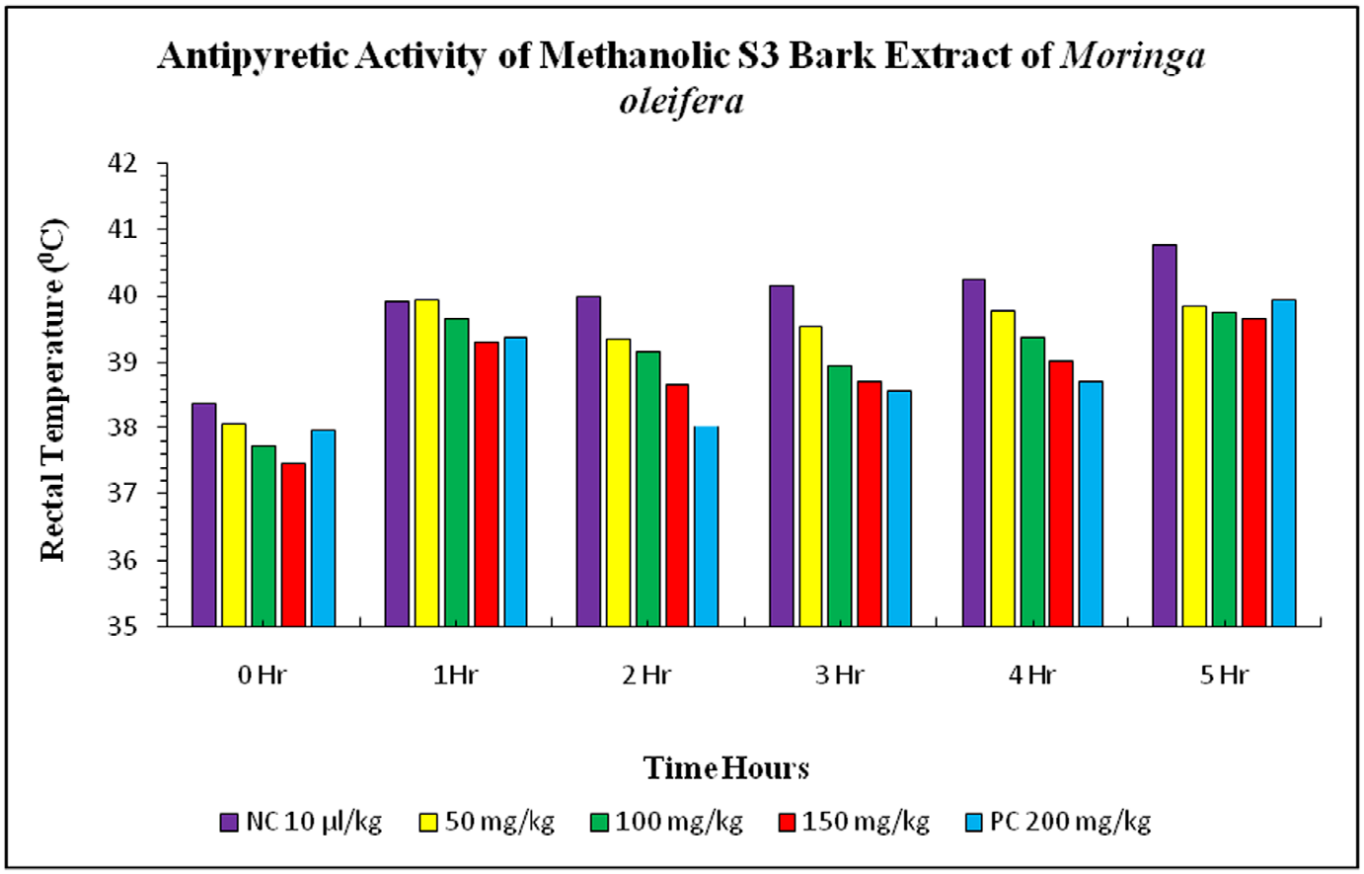
Antipyretic activity of S3 methanolic bark extract of *Moringa oleifera* on *Escherichia coli* ‐induced pyrexia in rabbits. NC = negative control, PC = positive control (paracetamol), S3 = Mianwali.

The study also investigated the effects of *M. oleifera'*s methanolic S1 bark extract on *E. coli* ‐induced pyrexia in rabbits. The animals' normal body temperature before the injection of *E. coli* suspension was 38.39°C ± 0.03°C. After 1 h of injection, the rectal temperature increased to 39.93°C ± 0.02°C in the negative control group and 39.38°C ± 0.02°C in the positive control group. The treatment groups showed a gradual decrease in rectal temperature, reaching 38.26°C ± 0.04°C in the 50 mg/kg treatment, 38.20°C ± 0.03°C in the 100 mg/kg treatment, and 38.12°C ± 0.06°C in the 150 mg/kg treatment. The maximum rectal temperature decreased in the 150 mg/kg treatment group compared to the negative and positive control groups (Figure 4). The study examined the effect of methanolic S2 bark extract of *M. oleifera* on *E. coli* induced pyrexia in rabbits. The normal body temperature of rabbits before the injection of *E. coli* suspension was 38.39°C ± 0.03°C and 37.96°C ± 0.02°C. The treatment groups of S2 bark extract at doses 50, 100, and 150 mg/kg showed a gradual increase in rectal temperature. After 1 h, the rectal temperature decreased with the doses applied. The rectal temperature increased with the doses at 50, 100, and 150 mg/kg treatments. The peak value of decrease in rectal temperature was recorded in the 150 mg/kg treatment group, which was closely observed in the treatment group with doses of 50 mg/kg and 100 mg/kg with *M. oleifera* (Figure 5). The study examined the effect of *M. oleifera* methanolic S3 bark extract on *E. coli* ‐induced febrile rabbits. The initial rectal temperature was 38.39°C ± 0.03°C before *E. coli* suspension injection. After 1 h, the *E. coli* suspension increased to 39.16°C ± 0.03°C in the negative control group and 39.38°C ± 0.02°C in the positive control group. However, the treatment group recorded higher temperatures at 50, 100, and 150 mg/kg. The methanolic S1 leaves extract showed a decrease in temperature after 3 h, but the treatment group's rectal temperature increased again. The treatment group's rectal temperature decreased after 4 h, but the treatment group's temperature increased again after 5 h (Figure 6).

**FIGURE 7:**
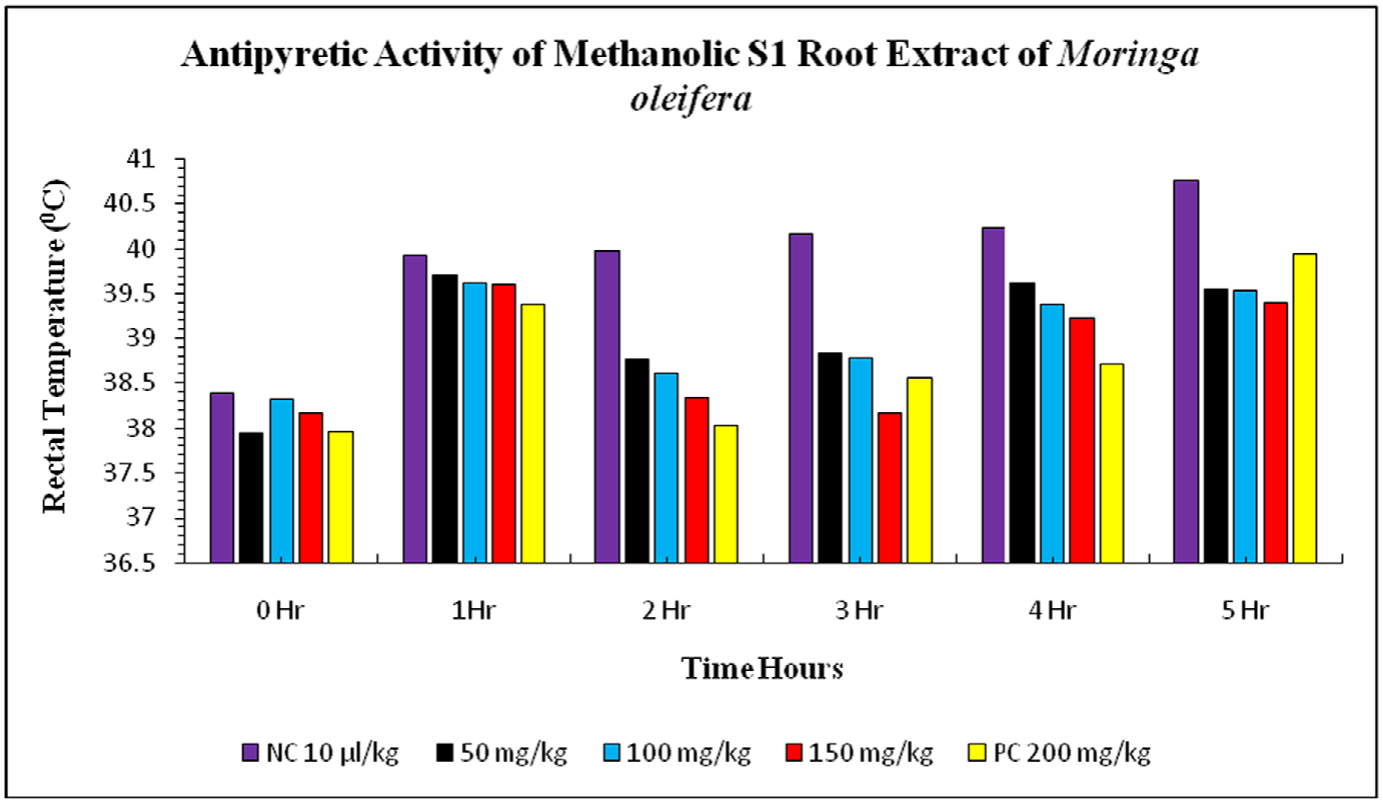
Antipyretic activity of S1 methanolic root extract of *Moringa oleifera* on *Escherichia coli‐*induced pyrexia in rabbits. NC = negative control, PC = positive control (paracetamol), S1 = Bahawalpur.

**FIGURE 8:**
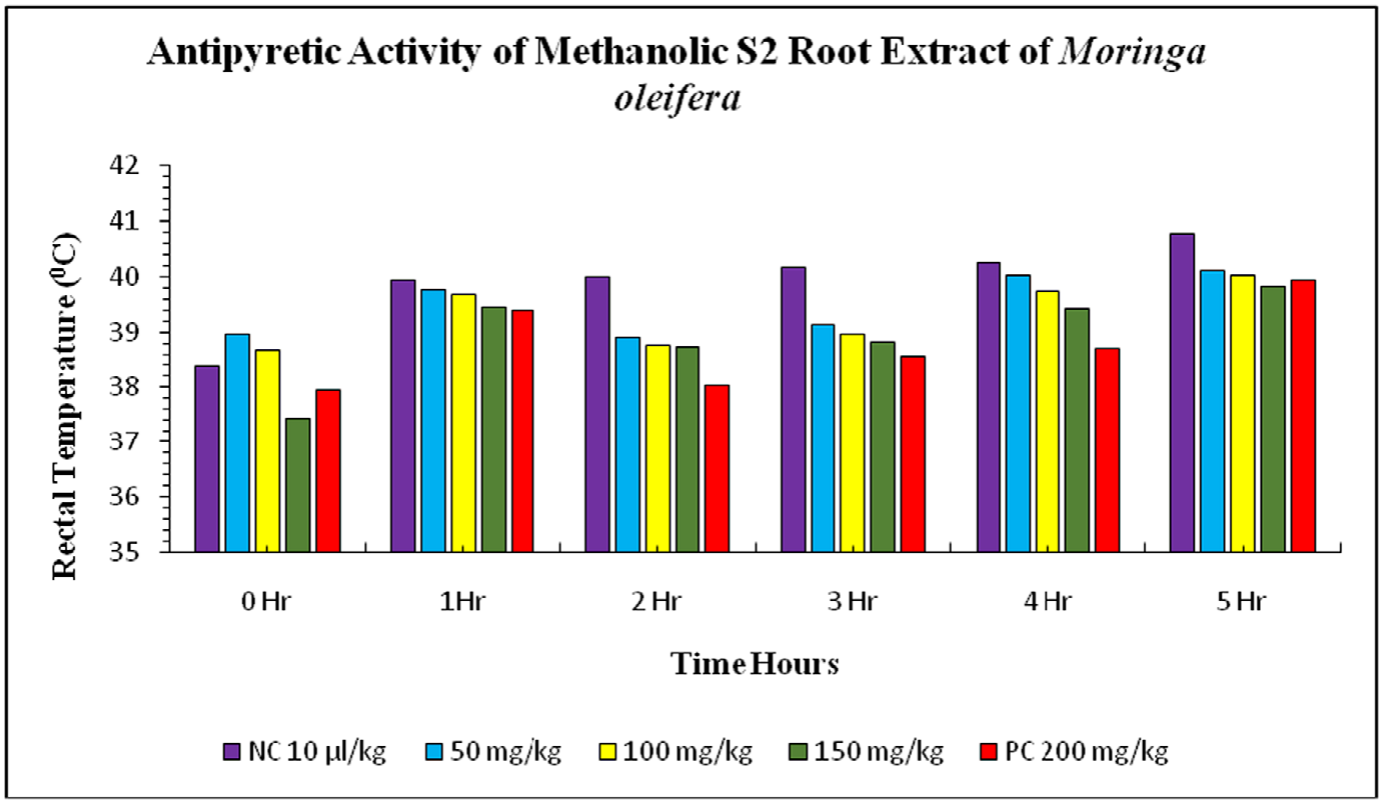
Antipyretic activity of S2 methanolic root extract of *Moringa oleifera* on *Escherichia coli‐* induced pyrexia in rabbits. NC = negative control, PC = positive control (paracetamol), S2 = Faisalabad.

**FIGURE 9:**
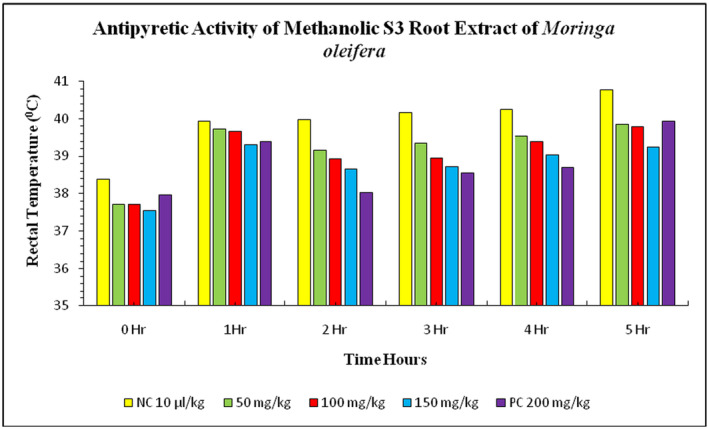
Antipyretic activity of S3 methanolic root extract of *Moringa oleifera* on *Escherichia coli‐*induced pyrexia in rabbits. NC = negative control, PC = positive control (paracetamol), S3 = Mianwali.

The study investigated the effect of methanolic S1 root extract of *Moringa* on rabbits' rectal temperatures. The normal body temperature of rabbits before *E. coli* suspension injection was 38.39°C ± 0.03°C, while the treatment group showed a significant decrease in rectal temperature at doses of 50, 100, and 150 mg/kg. After 1 hour of *E. coli* suspension injection, the rectal temperature was increased to 39.93°C ± 0.02°C in the negative control group and 39.38°C ± 0.02°C in the positive control group. The treatment group with methanolic S1 root extract showed a significant decrease in rectal temperature at doses of 50, 100, and 150 mg/kg. The highest level of decrease in rectal temperature was observed in the 150 mg/kg treatment group, closely followed by the 50 and 100 mg/kg treatments (Figure 7). The normal body temperature was 38.39°C ± 0.03°C before the injection of *E. coli* suspension in the negative control group (distilled water) and positive control group (paracetamol). After 1 h, the rectal temperature increased to 39.93°C ± 0.02°C in the negative control group and 39.38°C ± 0.02°C in the positive control group. The treatment groups showed a gradual decrease in rectal temperature, with the highest level of decrease observed in the 150 mg/kg treatment group. The highest level of decrease was observed in the treatment group with 50 and 100 mg/kg methanolic S3 leaves extract of *M. oleifera* (Figure 8). The treatment group of S3 root extract at doses 50, 100, and 150 mg/kg showed a gradual increase in rectal temperature. After 1 h, the rectal temperature decreased with the doses applied. The rectal temperature increased with the doses at 50, 100, and 150 mg/kg treatments. The peak value of decrease in rectal temperature was observed in the 150 mg/kg treatment group, which was closely plotted with the 50 and 100 mg/kg doses (Figure 9). The study highlights the potential of methanolic S1, S2, and S3 root extract in treating *E. coli* ‐induced pyrexia in rabbits. Current findings presented the phytochemical screening, significant variations in different nutritional and pharmacological activities in methanolic extract of leaves, bark, and root of *M. oleifera* from three different sites S1, S2, and S3. The leaves, bark, and root showed different potential of the studied activities among the S1, S2, and S3. The medicinal significance of the plants is associated with the presence of bioactive phytochemicals which have a specific physiological action on humans and can be used in treating numerous diseases (Jikah and Edo 2023). The highest moisture content was found in S3 leaves, followed by S2 leaves, and lowest moisture content in S1, that is the reason S1 leaves showed highest significance in every activity (Ogbe and Affiku 2011). Ash content was highest in S1, followed by S3. Crude fat, crude fiber, crude protein, and organic matter were also found to be significant in S1, S2, and S3. The highest organic matter content was found in S1, followed by S2 and S3, and the present findings also correlate the findings reported in the literature (Tafu and Jideani 2022). Rectal temperature dropped from 39.31°C to 38.66°C in the third treatment group at 150 mg/kg bw. Rectal temperature was 39.94°C in the positive control group (paracetamol) (also used by the scientist, Bhattacharya et al. 2014) at a dosage of 200 mg/kg, compared to 39.25°C in the treatment group at a dose of 150 mg/kg. The rectal temperature during drug treatment executed higher statistical difference (*p* < 0.05) at 1 h (Figure 9). The methanolic extract of *M. oleifera* leaves also showed the similar significant antipyretic activity (Hukkeri et al. 2006) as we found in our study. The findings of this study are also consistent with those of a number of other studies (Afsar et al. 2015; Hasan et al. 2022; Alyas et al. 2023).

Numerous investigations have documented the antipyretic and anti‐inflammatory potential of Moringa. The antipyretic effectiveness of *M. oleifera* leaves extract was reported in experimental albino rats, where it reduced the yeast‐induced pyrexia in a dose‐dependent manner maintaining the normal body temperature (Chhikara et al. 2021). A published study investigated a comparative analgesic potential of alcoholic extract of the leaves and seeds of Moringa between indomethacin and aspirin (Mabrok and Mohamed 2019).

## 4 Conclusion

The study concluded that the leaves of *M. oleifera* have highest nutritional content compared to the bark and roots. It was also found that S1 leaves, bark, and root extract of *M. oleifera* Lam demonstrated maximum antipyretic activity when compared to the S2 and S3 leaves, bark, and root extracts. The S1 leaves extract demonstrated outstanding antipyretic outcomes for reducing rectal temperature in rabbits.

## Author Contributions

**Shaista Jamil:** data curation (lead), methodology (lead), software (equal), writing – original draft (equal). **Tanveer Hussain Turabi:** conceptualization (lead), data curation (equal), formal analysis (equal), project administration (lead), resources (equal), supervision (lead). **Saeed Ahmad:** formal analysis (supporting), methodology (equal), visualization (equal), writing – original draft (equal). **Muhammad Riaz:** formal analysis (equal), validation (equal), visualization (lead), writing – review and editing (lead). **Hafiz Muhammad Wariss:** investigation (supporting), software (equal), visualization (lead), writing – review and editing (equal). **Quzi Sharmin Akter:** formal analysis (supporting), software (equal), visualization (equal), writing – review and editing (equal).

## Conflicts of Interest

The authors declare no conflicts of interest.

## Data Availability

The data will be available from the principal and corresponding authors on reasonable request.
